# Is It a Third Tooth, or the Development of the Long Dormant Pulp of a Second Molar?

**Published:** 1871-11

**Authors:** H. Scott


### IS IT A THIRD TOOTH, OR THE DEVELOPMENT OF THE LONG DORMANT PULP OF A SECOND MOLAR?

BY H. SCOTT.

A few days since, an old gentleman with whom I have been long acquainted, meeting me on the street, said, "*I have recently cut a new tooth.*" Presuming of course that the stump of some broken off molar had come to light below the gums, I invited him into a room that I might determine. I was mistaken. There was a large and perfectly formed molar on the left side of the upper jaw, occupying about the place of the second molar, though it was more like a first molar. The tooth appeared fresh and young, or about as the molar of a person of eighteen or twenty years.

Mr. M. is over sixty-five years of age. He told me he had nothing but stumps in his mouth for many years, and the last of them he had removed more than a year ago. The strange tooth came through the gums within the last two months, and developed rapidly.

